# Transformer-Based Semantic Segmentation for Extraction of Building Footprints from Very-High-Resolution Images

**DOI:** 10.3390/s23115166

**Published:** 2023-05-29

**Authors:** Jia Song, A-Xing Zhu, Yunqiang Zhu

**Affiliations:** 1 State Key Laboratory of Resources and Environmental Information System, Institute of Geographic Sciences and Natural Resources Research, Chinese Academy of Sciences, Beijing 100101, China; 2 Department of Geography, University of Wisconsin, Madison, WI 53706, USA; 3 Jiangsu Center for Collaborative Innovation in Geographical Information Resource Development and Application, Nanjing 210023, China

**Keywords:** vision transformer, hyperparameter, building, self-attention, deep learning

## Abstract

Semantic segmentation with deep learning networks has become an important approach to the extraction of objects from very high-resolution remote sensing images. Vision Transformer networks have shown significant improvements in performance compared to traditional convolutional neural networks (CNNs) in semantic segmentation. Vision Transformer networks have different architectures to CNNs. Image patches, linear embedding, and multi-head self-attention (MHSA) are several of the main hyperparameters. How we should configure them for the extraction of objects in VHR images and how they affect the accuracy of networks are topics that have not been sufficiently investigated. This article explores the role of vision Transformer networks in the extraction of building footprints from very-high-resolution (VHR) images. Transformer-based models with different hyperparameter values were designed and compared, and their impact on accuracy was analyzed. The results show that smaller image patches and higher-dimension embeddings result in better accuracy. In addition, the Transformer-based network is shown to be scalable and can be trained with general-scale graphics processing units (GPUs) with comparable model sizes and training times to convolutional neural networks while achieving higher accuracy. The study provides valuable insights into the potential of vision Transformer networks in object extraction using VHR images.

## 1. Introduction

Semantic segmentation is one of the key image classification tasks in the computer vision (CV) field. It is the process of classifying each pixel in an image belonging to a certain class and can be thought of as a classification problem per pixel [1,2]. In recent years, the success of semantic segmentation using deep convolutional neural networks (CNNs) has rapidly attracted research interest in the remote sensing community, and Object-based Image Analysis (OBIA) [3,4] has been transforming traditional image segmentation methods into semantic segmentation methods using CNNs [5,6,7,8,9,10]. CNN-based semantic segmentation is an efficient end-to-end learning approach to image classification at the pixel level [11,12,13]. With a large amount of training data, a CNN is able to automatically extract features from very-high-resolution (VHR) images obtained using aerial or satellite sensors and then apply them to extract natural or artificial objects [14,15,16,17] in VHR images. CNNs have been shown to perform better than swallow machine learning methods [18,19,20] and have become a dominant method in the extraction of objects from VHR images.

With the development of deep learning, a novel neural network architecture, Transformer [21], has garnered significant attention in the Natural Language Processing (NLP) field since 2017 [22,23,24], and efforts to develop Transformer networks for CV tasks have been promoted in recent years. Vision Transformer (ViT) [25], a vision model based as closely as possible on the Transformer architecture originally designed for text-based tasks, was proposed at the end of 2020. The notable highlight of Transformer is that it is the first model that relies entirely on a self-attention mechanism to capture the salient parts of input information, and this attention mechanism is one of the most valuable breakthroughs in deep learning in recent years [26,27]. The attention mechanism refers to the ability to dynamically highlight and use the salient parts of information [28], which is similar to the ability of the human brain to dynamically and instinctively select crucial information for decision-making. ViT attains excellent results compared to state-of-the-art CNNs, while it requires substantially fewer computational resources to train [29,30,31]. Additionally, in comparison with attention-enhanced CNN models, a pure Transformer applied directly to sequences of image patches without a CNN can perform very well in image classification tasks [18,32]; thus, this has been the inspiration for a new wave of vision Transformer networks [33,34], including Pyramid ViT [35], SegFormer [36], Swin Transformer [37], and so on.

Vision Transformer networks show great development potential in the field of computer vision. Nevertheless, investigations into Transformer-based networks for the extraction of geographical objects from VHR remote-sensing images remain scarce [38,39,40,41]. Vision Transformer networks have unique implementations such as image patches [42], linear embedding [43], and multi-head self-attention (MHSA) [44]. It remains unclear how we can more effectively configure them for the extraction of objects in VHR images and how they affect the accuracy of networks [45]. Therefore, this article leverages vision Transformer networks for object extraction from VHR remote-sensing images. As building footprints are essential artificial objects on the land surface and there are already several publicly available training datasets of buildings in the remote sensing classification community, we chose building footprints as the research object to investigate the impact on the accuracy of hyperparameters that are often specific to vision Transformer networks [46,47,48,49]. In the next section, existing studies on building footprint extraction methods are reviewed, and the foundations of vision Transformer networks are introduced to elucidate how vision Transformers work. In Section 3, a network based on Swin Transformer is presented for the extraction of building footprints. Based on this network, we set up eight different models, and each model had different Transformer-specific hyperparameter values. These models are presented in Section 4, as well as a comparison of the performance of each of them. Section 5 presents the experiment results, followed by a discussion of the results. Section 6 concludes this paper.

## 2. Related Work

### 2.1. Building Footprint Extraction Methods

Traditional building footprint extraction methods mainly rely on features designed manually by humans, such as the texture and geometric features of buildings, and the algorithms of building footprint extraction include the gray level co-occurrence matrix [50], Gabor wavelet transform [51], corner detection [52], and contour grouping [53]. However, due to the limited number of features and the model size, the deeper or more abstract features of building footprints are difficult to represent; thus, traditional building extraction methods usually have lower levels of extraction accuracy compared to deep learning methods.

With the advent of deep learning techniques, semantic segmentation methods based on convolutional neural networks (CNNs) have provided new approaches for the extraction of buildings from VHR images. These networks are mainly based on Fully Convolutional Networks (FCNs) [54], SegNet [55], U-Net [56], and DeepLab. For example, CNNs based on ResNet or DenseNet backbone networks combined with Conditional Random Fields (CRFs) [57], the U-Net++ network reconstructed with DenseNet as a backbone network [58], and the SegNet network improved with the Gaussian algorithm and Image Pyramid [59] are all CNN-based building footprint extraction methods. CNN-based methods have dominated the field of building footprint extraction for several years due to their ability to learn and extract complex features from VHR images.

In the last two years, with the great success of Transformer methods in the computer field, Transformer-based semantic segmentation methods have also been utilized for the extraction of building footprints [60,61,62,63], such as BuildFormer [64], a ViT-based model with a dual-path structure capable of capturing global context with large windows; MSST-Net [46], a multi-scale adaptive segmentation network model based on Swin Transformer; STT (Sparse Token Transformer) [29], an efficient dual-pathway Transformer structure that learns long-term dependencies in both spatial and channel dimensions; and STEB-UNet [65], a network integrating a Swin-Transformer-based encoding booster in a specially designed U-shaped network to achieve the feature-level fusion of local and large-scale semantics. These novel Transformer-based approaches show great promise for further improvements to the accuracy of building footprint extraction. However, it is important to note that the different hyperparameters of Transformers can also affect the model performance and should be considered. Therefore, this study pays more attention to the impact of the hyperparameters of the Swin Transformer, providing valuable insights into the more effective utilization of vision Transformer networks in VHR images.

### 2.2. Foundations of Transformers in Vision

**Figure 1:**
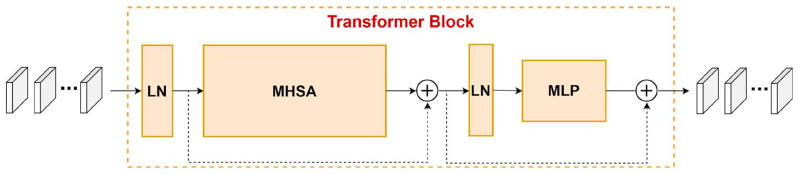
Framework of the Transformer block.

Transformers in vision are based on the architecture of the Transformer originally designed for text-based NLP tasks. Instead of a series of word embeddings as the inputs of the Transformer in NLP, image patches, which are generated via image partition, are the inputs of Transformers in vision, and the attention is computed on top of the image patches. Transformers in vision consist of a stack of Transformer blocks, and the Transformer block includes Layer Normalization (LN), multi-head attention (MHA), and Multi-layer Perceptron (MLP), as shown in Figure 1. Residual connections are applied on both MHSA and MLP to resolve the difficulty in the convergence of multi-layer neural networks.

### 2.3. Layer Normalization (LN)

**Figure 2:**
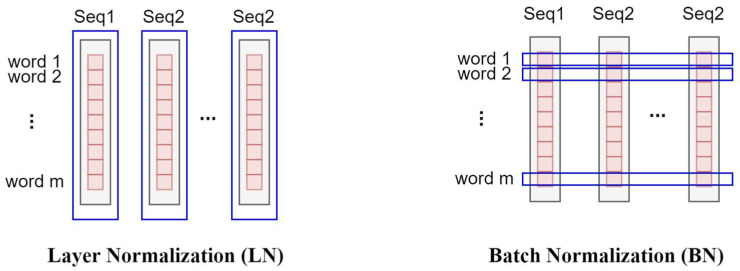
Layer Normalization (LN) and Batch Normalization (BN).

LN is used before every block and residual connections after every block in a Transformer to scale the features for each sample of a sequence. LN helps to speed up and stabilize the learning process. Additionally, LN [66] is proven to yield significantly better performance than Batch Normalization (BN) in Transformers, and BN is often used in CNNs to scale an entire feature map. For a batch of sentences in Transformers in NLP, BN scales over the words at the same position of each sentence, and LN scales over all the words in each sentence, as shown in Figure 2. Obviously, scaling the words at the same position in different sentences does not follow the design of sequence models, whereas LN satisfies the requirements of Transformers.

### 2.4. Multi-Head Self Attention (MSA)

**Figure 3:**
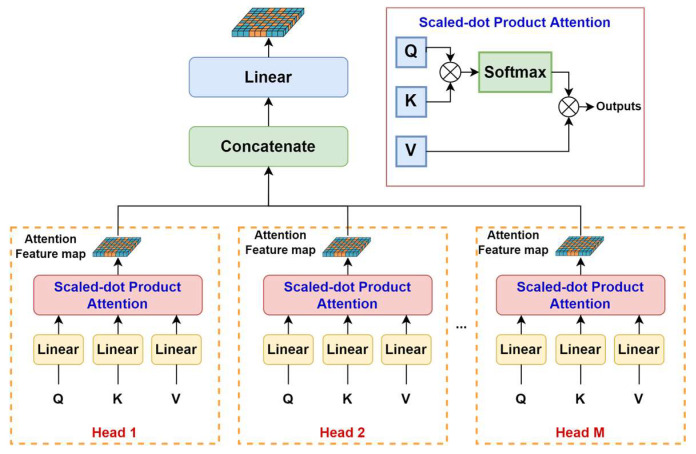
Standard multi-head self-attention in Transformer.

MSA in Transformers is multiple self-attentions in parallel, and each head of self-attention is concatenated and then projected to outputs, as shown in Figure 3. Most Transformers use standard self-attention [21], which is based on scaled-dot products to compute self-attention. Three inputs of Queries (*Q*), Keys (*K*), and Values (*V*) are used to generate self-attention feature maps. *Q* and *K* are used to generate weights of features, and the weights work on *V*, generating self-attention feature maps. The *Q*, *K*, and *V* of standard self-attention are outputs of linear operations with the learnable parameters *W^Q^*, *W^K^*, and *W^V^*, and the standard self-attention is computed as:

(1) $$SelfAttention\left(Q,\;K,\;V\right)=Softmax\left(\frac{QK^{T}}{\sqrt{d_{k}}}\right)V$$

where *d_k_* is the dimension of both *Q* and *K*, and the Softmax function scales the weights in the range [0, 1] and makes the weights equal to one. The multi-head self-attention links multiple convolution kernels in CNNs to generate multiple feature maps. The more self-attention feature maps there are, the better the performance models could achieve. The multi-head self-attention is computed as:

(2) $$MSA\left(Q,\;K,\;V\right)=Concat\left(head_{1},\;\ldots ,\;head_{h}\right)W^{O}$$

where $head_{i}=SelfAttention(QW_{i}^{Q},\;KW_{i}^{K},\;VW_{i}^{V})$.

### 2.5. Multi-Layer Perceptron (MLP)

MLP, also known as the Feed-Forward network (FFN), consists of two linear layers and a GELU nonlinearity in Transformers. The outputs from MLP are added to the inputs (skip connection) to obtain the final output of the Transformer block. The role and purpose of MLP are to process the output from one attention layer in a way that fits the input for the next attention layer better.

## 3. Transformer-Based Network for Extraction of Build Footprints from VHR Images

### 3.1. Network Architecture

**Figure 4:**
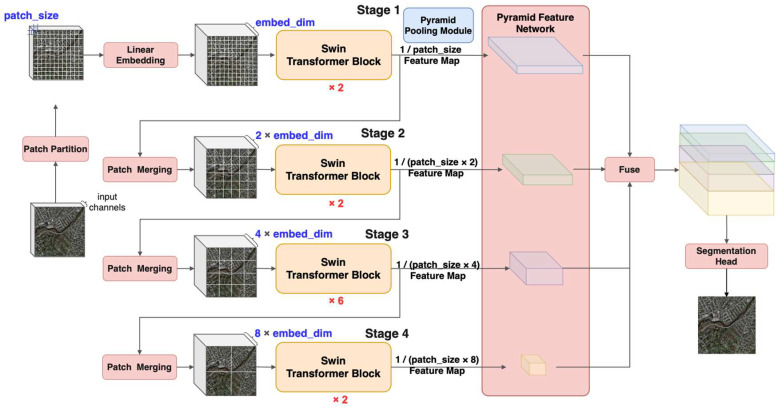
The structure of the Transformer-based network for extraction of building footprints from VHR images.

The proposed Transformer-based network for building extraction has an encoder-decoder architecture, as shown in Figure 4. A novel Swin Transformer is utilized as the encoder to extract the multi-scale-self-attention-based features of the VHR images. Based on the multi-scale features, we further introduce a Pyramid Pooling Module (PPM) [67] in the decoder to add global context to a VHR image; then, we use a Feature Pyramid Network (FPN) [68] in the decoder to fuse the multiple different scales of feature maps. All of these fused feature maps are upsampled into the original resolution of the VHR image via a segmentation head. The segmentation head projects the feature maps onto the pixel space to obtain pixel-by-pixel coverage of the building footprints.

### 3.2. Network Modules

The proposed network is composed of a Patch Partition module, Linear Embedding module, Patch Merging module, Swin Transformer block module, Pyramid Pooling Module, and Feature Pyramid Fusion Module. They are described as follows:

#### 3.2.1. Patch Partition

The Patch Partition module is the first layer of the Transformer-based encoder. The Patch Partition Layer splits the raw VHR image into non-overlapping patches for the application of self-attention to the image patches rather than the pixels. The self-attention to image patches can reduce the time complexity of training and thus make the Transformer-based network applicable to a large number of VHR images.

#### 3.2.2. Linear Embedding

“Embedding” means taking some sets of raw inputs and converting them to vectors in machine learning. The Linear Embedding module in Vision Transformers thus takes a sequence of image patches as the input and generates a vector representation of the image patches in another mathematical space using a linear transformation. It can be seen as the abstract representation of the original information at the semantic level. Additionally, with the Linear Embedding module, the arbitrary channel number and arbitrary size of image patches can be transformed into a sequence of one-dimension vectors with the same length, thereby enhancing the model’s ability to adapt to different kinds of images as inputs.

#### 3.2.3. Swin Transformer Block

**Figure 5:**
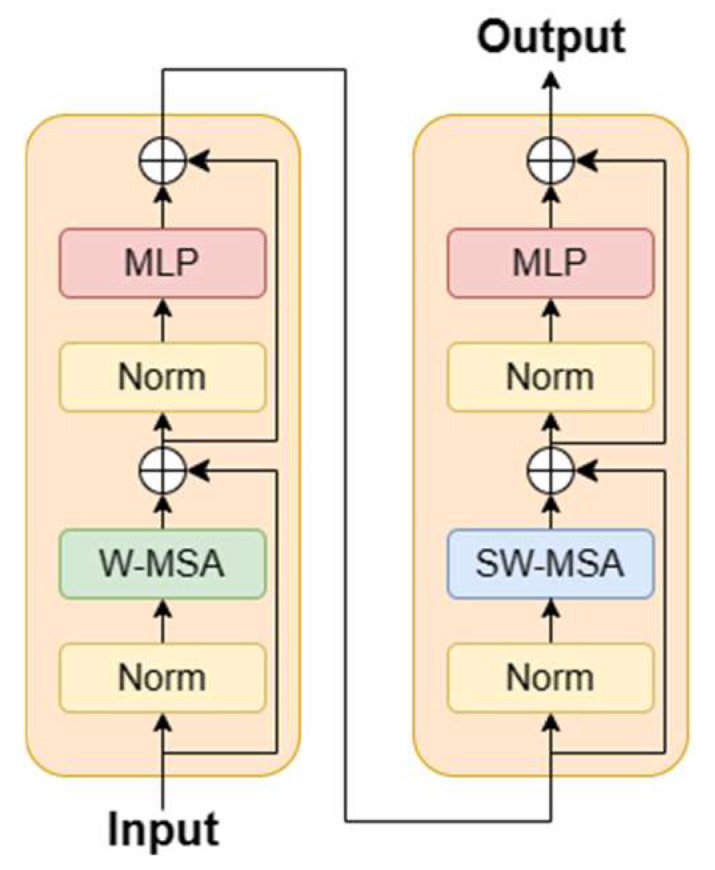
Swin Transformer block.

Swin Transformer blocks [37] are kernels in the Transformer-based building extraction network which implement the self-attention mechanism in an efficient way. Swin Transformer blocks are often stacked to capture deeper and more advanced features, as CNN blocks do. Inside a Swin Transformer block, a shifted window is introduced to compute both local and global self-attention. The shifted windows are non-overlapping windows that partition the VHR images on the top of image patches. To reduce quadratic complexity in computing self-attention, two successive Swin Transformer blocks can achieve self-attention computation with less complexity, as shown in Figure 5. The first Swin Transformer block contains a window-based multi-head attention (W-MSA) module which computes the self-attention within the window, and the second Swin Transformer block contains a shifted-window-based multi-head attention (SW-MSA) module, which computes self-attention across the windows by alternating between two partitioning configurationsW in consecutive Swin Transformer blocks. Therefore, two successive Swin Transformer blocks can compute the self-attention computation over the whole VHR image, and the computation takes less time.

#### 3.2.4. Pyramid Pooling

To make the model learn not only the detailed features but also the global features of VHR images, we introduce the Pyramid Pooling module in the decoder to capture the global context of the feature map learned by the encoder. The Pyramid Pooling module is an effective global prior representation and captures the global context using a CNN-based multi-level pyramid. Each level of the multi-level pyramid is a pooling layer with a different pooling rate. A multi-level pyramid of pooling layers can learn different granularities of global features, which enables the model to more comprehensively grasp information regarding the global scene of VHR images.

#### 3.2.5. Feature Pyramid Fusion

To more effectively utilize the multi-scale feature maps generated by the encoder, the Feature Pyramid Fusion (FPN) module is applied in the decoder to fuse the feature maps from the Pyramid Pooling and the Swin Transformer block. With the FPN, feature maps with different sizes and channel numbers are fused to a single feature map. The fused feature map integrates all the features at different levels and thus may help to further improve the classification accuracy.

### 3.3. Transformer-Specific Hyperparameters

The main Transformer-specific hyperparameters in the network are patch size, embedding dimension, and window size. They are described as follows:

(1) Patch size

The patch size refers to the size of image patches and determines how many pixels are in a unit to generate feature maps based on the self-attention calculation method. The patch size is related to the resolution of the feature maps. When a VHR image is represented as

(3) $$\;Image\left(X\right)\in \;R^{H\times W\times C}$$

where H,  W is the height and width of the VHR image, and C is the channel of the VHR image, the VHR image patches can be represented as

(4) $$ImagePatches\left(X\right)\in \;R^{\left(P\times P\times C\right)\times N}$$

where P is the patch size, and N is the length of the sequence of image patches ($N=H\times W/\;P^{2}$). Each patch is flattened to a vector with a length of P×P×C  before it is passed into the Linear Embedding module.

(2) Dimension of embeddings

The dimension of embeddings refers to the length of a vector that represents an embedded image patch. The embedded image patches are generated by the Linear Embedding module, represented as

(5) $$PatchEmbeddings\left(X\right)\;\in \;R^{D\times N}$$

where D is the dimension of embeddings, and *N* is the length of the sequence of embeddings, which is the same as the length of image patches.

(3) Window size

The window size refers to how many image patches are grouped to directly calculate self-attention within windows; thus, a larger window size means more image patches are used to directly calculate the window-level self-attention. Supposing each window contains M × M patches, the feature map generated by the Swin Transformer block in Stage 1 is represented as

(6) $$FeatureMap_{stage1}\left(X\right)\in \;R^{H/P\times W/P\times D\;}$$

After merging image patches, the feature maps generated by the Swin Transformer block in Stage 2, Stage 3, and Stage 4 are represented as

(7) $$FeatureMap_{stage2}\left(X\right)\in \;R^{H/2P\times W/2P\times 2D\;}$$

(8) $$FeatureMap_{stage3}\left(X\right)\in \;R^{H/4P\times W/4P\times 4D\;}$$

(9) $$FeatureMap_{stage4}\left(X\right)\in \;R^{H/8P\times W/8P\times 8D\;}$$

## 4. Experimental Section

### 4.1. Datasets

We chose the publicly available Massachusetts Buildings Dataset (https://www.cs.toronto.edu/~vmnih/data/ accessed on 1 September 2022) as the experiment data. The Massachusetts Buildings Dataset consists of 151 aerial images in the Boston area of the U.S. Each image is 1500 pixels × 1500 pixels with red, green, and blue bands, and the spatial resolution is 1 m. The original 151 images were split into a training dataset of 137 images, a validation dataset of 4 images, and a test dataset of 10 images.

Due to the limitation of GPU memory, the original 1500 pixel × 1500 pixel images needed to be divided into smaller images in the experiment. Due to the hierarchical structure of the Swin Transformer, the arbitrary sizing of image samples is not recommended. The downscaling was performed during the generation of the multi-scale attention-based feature maps, and the upscaling was performed when merging them. Hence, inappropriate image sizes will lead to merging failure in the Swin Transformer. By analyzing the structure of this network, the appropriate image size was determined to be $patch\_size\times 2^{merge\_times}\times window\_size\;$ or the integer multiples of it, and Table 1 lists the appropriate image sizes between approximately 200 and 400 pixels. The patch number in the Nth stage was calculated using H/P/N × W/P/N, where H and W are the height and width of images, P is the size of the image patches, and N is the sequential number of the stages (i.e., 1, 2, 3, 4). Considering maximization by utilizing the original 1500 pixels × 1500 pixels images, the image size selected in this experiment was 288 × 288 pixels. Therefore, we finally obtained 3000 pieces of 288 × 288-pixel samples for training and 98 pieces of the same-sized samples for validation.

### 4.2. Hyperparameter Settings

In order to explore how the Transformer-specific hyperparameters affect the accuracy of the extraction of building footprints, we set up eight experiment groups, and each group had different Transformer-specific hyperparameter values, as shown in Table 2. They all were trained by the same training samples described in Section 4.1.

### 4.3. Training Settings

The eight building footprint extraction networks with the different hyperparameter values were trained on the same NVIDIA GeForce RTX 3080 Ti GPU with 12 GB memory for 200 epochs. The batch size of the training samples was set to four due to the capacity limitation of GPU memory. The optimizer employed in the experiment was AdamW, with an initial learning rate of 6 × 10^−5^ and a weight decay of 0.01. In addition, a scheduler of linear learning rate was used to train the models with a warmup of 10 iterations. The building footprint extraction networks were not pre-trained on any other datasets, and no data augmentation methods were applied.

### 4.4. Evaluation Metrics

Four evaluation metrics were used in this study to evaluate the inference results. They are listed as follows:

(1) Overall accuracy (OA)

Accuracy is the metric calculated in the simplest way. It is the ratio of the correct predictions to the total number of predictions, represented as

(10) $$OA=\frac{TP+TN\;}{TP+TN+FP+FN}$$

where TP, FP, TN, and FP are the number of true positives, false positives, true negatives, and false negatives, respectively, in the confusion matrix;

(2) Intersection over union (IoU)

The mIoU is the average IoU. The IoU, also known as the intersection over union, is often used in object detection and semantic segmentation. It is the ratio of the overlap and union areas of prediction and ground truth. The mIoU can also be represented as

(11) $$\;mIoU=\frac{1}{n+1}\overset{n}{\underset{i=1}{\sum }}\;\;\frac{TP\;}{TP+FP+FN}$$

(3) F1-score

The F1-score is a metric that combines the precision and recall metrics, and it is more suitable for imbalanced data. The F1 score is defined as the harmonic mean of precision and recall, represented as

(12) $$F1\;score=2\times \frac{Precision\times Recall\;}{Precision+Recall}$$

(4) Kappa

Kappa, also known as Cohen’s Kappa [69], is a metric used to assess the agreement between two raters. Kappa is also a useful evaluation metric when dealing with imbalanced data. It is represented as

(13) $$\;Kappa=\frac{p_{0}-p_{e}\;}{1-p_{e}}$$

where $p_{0}$ is the overall accuracy of a model and $p_{e}$ is the measure of the agreement between the model predictions and the ground truth values.

## 5. Results and Discussion

### 5.1. Accuracy Evaluation

Accuracy evaluation was performed when training the models. After every epoch, the evaluation was performed using the validation samples described in Section 4.1.

**Figure 6:**
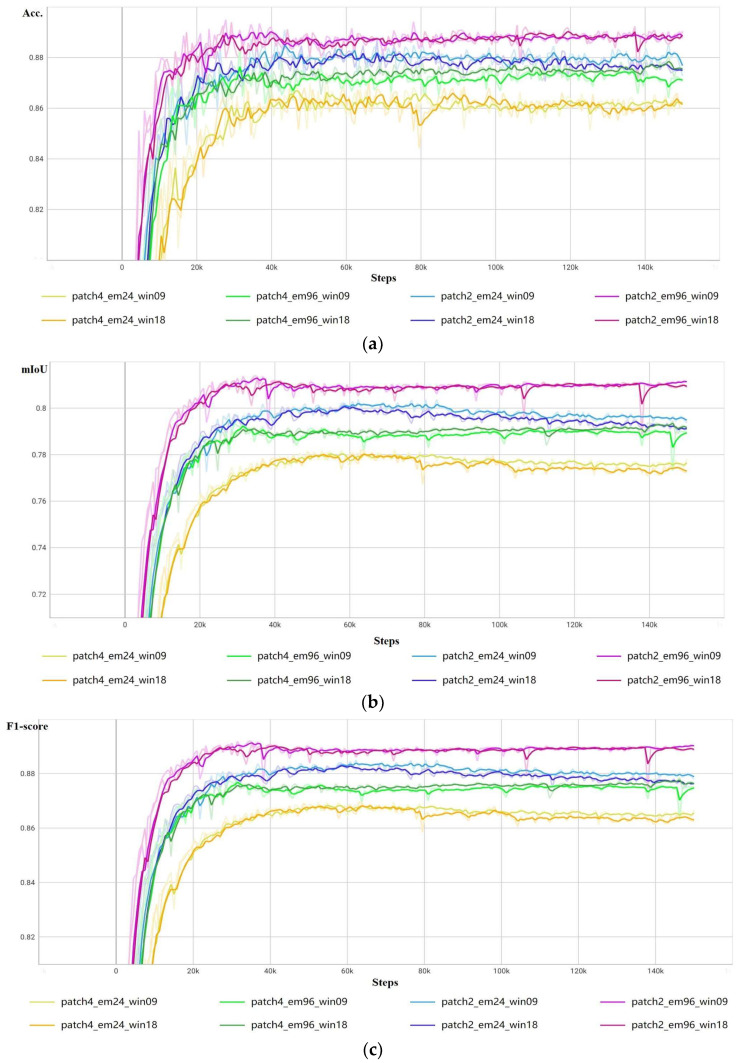

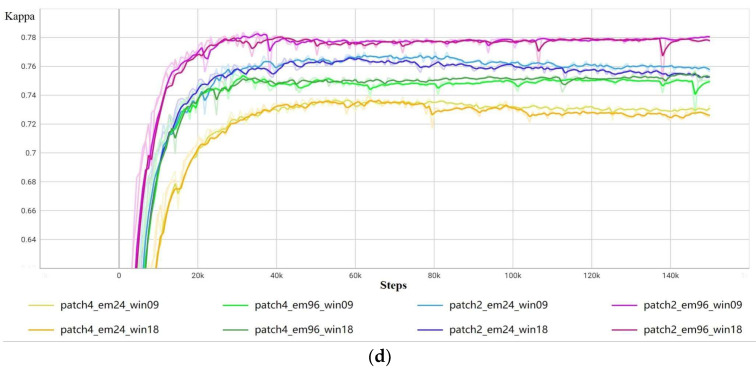
The accuracy evaluation curves on the validation samples. (**a**) OA change curve on the validation dataset. (**b**) mIoU change curve on the validation dataset. (**c**) F1-score change curve on the validation dataset. (**d**) Kappa change curve on the validation dataset.

Figure 6 shows the accuracy variation curve on the validation samples during training. It demonstrates that the networks with the 2 × 2 pixels image patches and 96-dimensional embeddings (i.e., ‘patch2_em96_win09’ and ‘patch2_em96_win18’) achieved the highest score for all metrics. Figure 5 also demonstrates that when the patch size and the dimension of the embeddings of the build footprint extraction networks were the same, the varying curves of OA, mIoU, F1-score, and Kappa were very similar, which indicates that the window size of the network has little impact on the accuracy of the building footprint extraction.

Table 3 further lists the top-three accuracy evaluation results. The ‘patch2_em96_win09’ experiment group achieved the best performance, which comprised values of 0.8913 for OA, 0.8138 for mIoU, 0.8919 for F1-score, and 0.7838 for Kappa, and the ‘patch2_em96_win18’ experiment group had very similar evaluation results to ‘patch2_em96_win09’. The ‘patch2_em96_win09’ and ‘patch2_em96_win09’ experiment groups had the same 96-dimension embeddings and two-pixel-sized image patches, and only their window sizes were different. Table 3 also demonstrates that the other experiment groups, which had the same patch sizes and embedding dimensions but different window sizes, had similar evaluation results. For example, ‘patch4_em24_win09’ and ‘patch4_em24_win18’ had the same four-pixel-sized image patches and 24-dim embeddings, and their results were similar.

Table 3 also shows that the vision Transformer networks with 96-dim embeddings had higher levels of evaluation accuracy than those with 24-dim embeddings. Higher-dimensional embeddings can represent richer features of buildings on remote sensing images. With the representation of richer features, the network can more effectively distinguish buildings from other objects, thereby obtaining a higher level of accuracy. Additionally, as the dimension of embeddings reflects the level of feature representation, we suggest higher-dimensional embeddings are suitable for extracting features of complex objects such as crops and wetlands, while lower dimensions can be relatively simple objects such as water and ice. However, it should be noted that using higher-dimensional embeddings increases the size of the model, resulting in higher CPU and GPU memory usage. Hence, given high-dimensional embeddings, it is necessary to pay attention to the size of the model so as not to exceed GPU memory limitations.

Table 3 also shows that the vision Transformer networks with two-pixel-sized image patches outperformed those with four-pixel-sized image patches, as image patches, rather than pixels, are used to calculate self-attention in vision Transformer networks. Smaller image patches generate higher-resolution features that are fed into the model and calculated to output attention feature maps. A finer attention feature map obviously reduces the number of errors raised by upsampling to the original size of images. As a result, using smaller image patches improves the accuracy of building footprint extraction. Also, unlike the common use of four- or six-pixel-sized patches in natural images, our findings indicate that two-pixel-sized patches are preferred in the context of VHR image analysis. Therefore, we recommend using two-pixel-sized patches for building footprint extraction tasks to maximize accuracy and reduce errors related to upsampling.

In addition, we compared Transformer-based methods to the CNN-based methods in the extraction of building footprints, as shown in Table 4. U-Net and DeepLab V3 are the most commonly used networks in the extraction of building footprints; thus, they were selected for comparison. Table 4 shows that the Transformer-based network outperformed the CNN-based U-Net and DeepLab V3 networks in all of the evaluated metrics. This result is consistent with the result in the CV field.

In general, the accuracy evaluation results confirm that the size of image patches and the dimension of embeddings has significant impacts on the accuracy of the extraction of building footprints using vision Transformer networks. Smaller-sized image patches or higher-dimension embeddings can achieve a higher level of accuracy in building footprint extraction, whereas the parameter of window size has little impact on the accuracy.

### 5.2. Model Size and Training Time

With the same GPU, the training time is mainly determined by the size of the model and training samples. In this study, the number of training samples was 3000, and the size of each sample was 288 × 288 pixels. The training times for the eight experiment groups are listed in Table 5. It can be seen that, in general, the training time of our Transformer-based building footprint extraction network was approximately between 9 and 12 h. The exact training time for each experimental group was slightly different due to the different parameter settings. We can see that the higher the embedding dimension was, the longer the training took since higher-dimensional embeddings lead to larger models.

### 5.3. Prediction Results

Since buildings in remote sensing images have different sizes and non-buildings could be misclassified as buildings, we show the prediction results in terms of large buildings, small buildings, and non-building misclassification.

**Figure 7:**
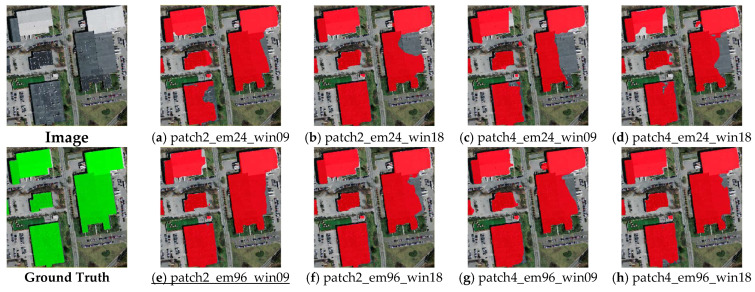
Example of prediction results for large buildings (underline denotes the best result among (**a**–**h**)).

**Large buildings**. In this study, buildings with areas larger than 1000 sq. meters were classified as large buildings, such as shopping malls, big libraries, and museums. Figure 7 shows the results of the large building footprints predicted using the Transformer-based building footprint extraction network. It demonstrates that the models with 96-dim embeddings (i.e., Figure 7e–h) generally outperformed the ones with 24-dim embeddings (i.e., Figure 7a–d), and the integrity of the large building footprint boundaries extracted using the 96-dim embeddings was better than that of those extracted using the 24-dim embeddings. We believe that higher-dimensional embeddings have more parameters, which helps to more accurately represent the overall characteristics of large buildings, resulting in better integrity when extracting them. Regarding the patch size, the results show its value was less sensitive than the embedding dimensions to the large buildings. This demonstrates that patch size is related to spatial resolution, and spatial resolution has a small impact on the extraction of large buildings from VHR images.

**Figure 8:**
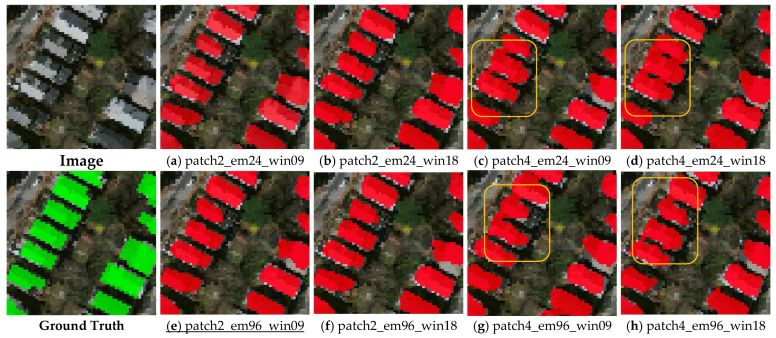
Example of prediction results for small buildings (underline denotes the best result among (**a**–**h**), and the overlapping area is circled in orange).

**Small buildings.** In this study, buildings with areas smaller than 300 sq. meters were classified as small buildings, such as houses and small commercial buildings. Figure 8 shows the results of the small building footprints predicted using the Transformer-based building footprint extraction network. The results demonstrate that the models with 2 × 2-pixel image patches (i.e., Figure 8a,b,e,f) generally outperformed those with 4 × 4-pixel image patches (i.e., Figure 8c,d,g,h). This suggests that smaller image patches are more effective for the prediction of the footprints of small buildings. These results could be explained by the fact that using smaller image patches helps the network capture finer details and edges, which can be important for the accurate prediction of small buildings’ footprints. In contrast, using larger image patches may result in the loss of some finer details, as well as the overlapping of the extracted building footprints.

**Figure 9:**
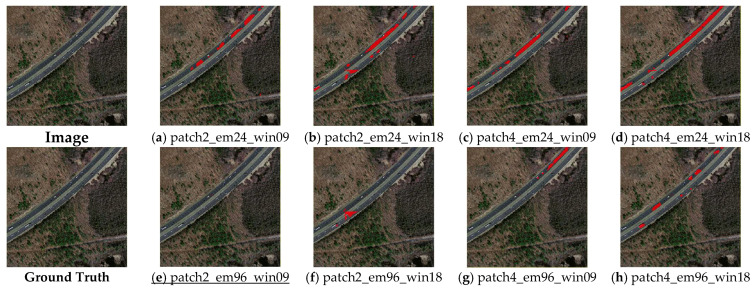
Example of the misclassified result of roads (underline denotes the best result among (**a**–**h**)).

**Non-building misclassification.** In this study, the main non-building objects misclassified as building footprints are roads. Figure 9 shows an example of roads being misclassified as building footprints. It can be seen that the ‘patch2_em96_win09’ experiment group (i.e., Figure 9a) achieved the best performance, and a few pixels of roads were misclassified as building footprints. The ‘patch2_em96_win09’ experiment group, which was only different in terms of window size, only misclassified a few pixels of roads. Figure 6 also demonstrates the models with 24-dim embeddings (i.e., Figure 9a–d) misclassified roads more seriously than the models with 96-dim embeddings (i.e., Figure 9e–h), especially for the models with patch sizes of 4 × 4 pixels (i.e., Figure 9c,d).

## 6. Conclusions

Vision Transformer networks have been developed as an alternative to CNNs and have shown significant improvements in performance over traditional CNNs in multiple tasks such as image classification, object detection, and semantic segmentation. This study explored the potential of vision Transformer networks in extracting geographical objects from VHR images, with a focus on building footprints. Moreover, we analyzed the particular hyperparameters of Swin Transformer networks, such as image patches, linear embedding, and window size, and investigated how they affect the accuracy of the extraction of building footprints. We found the hyperparameters of image patches and linear embedding had significant impacts on the accuracy. Smaller image patches resulted in higher accuracy in building footprint extraction. High-dimensional embeddings also resulted in higher accuracy in building footprint extraction. The window size had a smaller impact on the accuracy, but it impacted the size of the model, thereby affecting the training time. With the same image patches and embeddings, we recommend a smaller window size for the Swin Transformer network. These results provide an essential reference in Transformer-based network hyperparameter configuration to improve the accuracy of land cover classification with VHR images. In our experiment, when the size of the image patches was 2 × 2 pixels, the dimension of the embeddings was 96, and the window size was nine, the network achieved the highest accuracy in building footprint extraction. The values were 0.8913 for OA, 0.8138 for mIoU, 0.8919 for F1-score, and 0.7838 for Kappa, and the accuracy evaluation was based on the Massachusetts Buildings Dataset (https://www.cs.toronto.edu/~vmnih/data/ accessed on 16 November 2022). In addition, the experiment also showed that the Swin Transformer network could be trained with general-scale GPUs when applying VHR remote sensing images, and the model size and training time are acceptable compared to traditional CNNs while achieving better accuracy. This further demonstrates that Transformer networks are highly scalable and have broad potential applications in the field of remote sensing.

## Figures and Tables

**Table 1:** Appropriate image sizes between approximately 200 and 400 pixels.

Input Image Size (Pixels)	Utilization for the Original 1500 × 1500 Pixel Image	Patch Size (Pixels)	Patch Numbers of the Four Stages	Window Size (Patches)
224	89.6%	2	[112^2^, 56^2^, 28^2^, 14^2^]	7 or 14
		4	[56^2^, 28^2^, 14^2^, 7^2^]	7
256	85.3%	2	[128^2^, 64^2^, 32^2^, 16^2^]	8 or 16
		4	[64^2^, 32^2^, 16^2^, 8^2^]	8
288	**96.0%**	2	[144^2^, 72^2^, 36^2^, 18^2^]	9 or 18
		4	[72^2^, 36^2^, 18^2^, 9^2^]	9
320	93.9%	2	[160^2^, 80^2^, 40^2^, 20^2^]	10 or 20
		4	[80^2^, 40^2^, 20^2^, 10^2^]	10
352	93.9%	2	[176^2^, 88^2^, 44^2^, 22^2^]	11 or 22
		4	[88^2^, 44^2^, 22^2^, 11^2^]	11
384	76.8%	2	[192^2^, 96^2^, 48^2^, 24^2^]	12 or 24
		4	[96^2^, 48^2^, 24^2^, 12^2^]	12

Note: the largest utilization is highlighted in bold.

**Table 2:** Experiment group for the Transformer-specific hyperparameter.

Experiment Group	Patch Size (Pixels)	Embedding Dimension	Window Size (Patches)
patch2_em24_win09	2	24	9
patch2_em96_win09	2	96	9
patch2_em24_win18	2	24	18
patch2_em96_win18	2	96	18
patch4_em24_win09	4	24	9
patch4_em96_win09	4	96	9
patch4_em24_win18	4	24	18
patch4_em96_win18	4	96	18

**Table 3:** Evaluation metrics scores for the different models.

Experiment Group	Top-3	OA	mIoU	F1-Score	Kappa	Epoch
patch4_em24_win09	1	0.8663	0.7822	0.8696	0.7393	96
	2	0.8675	0.7821	0.8695	0.7391	85
	3	0.8627	0.7820	0.8694	0.7388	77
	**Avg**	**0.8655**	**0.7821**	**0.8695**	**0.7391**	**-**
patch4_em24_win18	1	0.8673	0.7822	0.8697	0.7393	84
	2	0.8642	0.7815	0.8691	0.7382	85
	3	0.8682	0.7814	0.8691	0.7382	65
	**Avg**	**0.8666**	**0.7817**	**0.8693**	**0.7386**	**-**
patch4_em96_win09	1	0.8838	0.7945	0.8785	0.7569	42
	2	0.8743	0.7933	0.8775	0.7550	39
	3	0.8730	0.7925	0.8769	0.7538	40
	**Avg**	**0.8770**	**0.7934**	**0.8776**	**0.7552**	**-**
patch4_em96_win18	1	0.8804	0.7947	0.8786	0.7571	195
	2	0.8775	0.7937	0.8778	0.7557	186
	3	0.8782	0.7936	0.8778	0.7555	189
	**Avg**	**0.8787**	**0.7940**	**0.8781**	**0.7561**	**-**
patch2_em24_win09	1	0.8864	0.8038	0.8851	0.7703	107
	2	0.8818	0.8038	0.8850	0.7701	111
	3	0.8855	0.8037	0.8850	0.7700	83
	**Avg**	**0.8846**	**0.8038**	**0.8850**	**0.7701**	**-**
patch2_em24_win18	1	0.8802	0.8019	0.8837	0.7674	78
	2	0.8827	0.8013	0.8834	0.7668	81
	3	0.8872	0.8011	0.8833	0.7666	91
	**Avg**	**0.8834**	**0.8014**	**0.8835**	**0.7669**	**-**
patch2_em96_win09	1	0.8931	0.8139	0.8920	0.7839	46
	2	0.8909	0.8138	0.8919	0.7837	42
	3	0.8898	0.8138	0.8918	0.7837	47
	**Avg**	**0.8913**	**0.8138**	**0.8919**	**0.7838**	**-**
patch2_em96_win18	1	0.8900	0.8124	0.8909	0.7819	47
	2	0.8898	0.8121	0.8907	0.7814	53
	3	0.8940	0.8119	0.8906	0.7813	39
	**Avg**	**0.8913**	**0.8121**	**0.8907**	**0.7815**	**-**

Note: underline denotes the highest score.

**Table 4:** Quantitative comparison with CNN-based methods.

Methods	Parameters (Million)	OA	mIoU	F1-Score	Kappa
U-Net	7.7	0.8271	0.7412	0.8390	0.6780
DeepLabV3	39.6	0.8339	0.7471	0.8440	0.6881
Swin Transformer+PFN (patch2_em96_win09)	62.3	0.8913	0.8138	0.8919	0.7838

**Table 5:** Training time, model parameters, and sizes.

Experiment Group	Training Time (Hours)	Model Parameters (Million)	Model Size (MB)
patch4_em24_win09	8.8	8.9	35
patch2_em24_win09	9.0		
patch4_em24_win18	9.4	8.9	35.6
patch2_em24_win18	9.8		
patch4_em96_win09	10.4	62.3	249.2
patch2_em96_win09	10.8		
patch4_em96_win18	12.4	62.3	249.2
patch2_em96_win18	12.9		

## Data Availability

The original Massachusetts Buildings Dataset is available at (https://www.cs.toronto.edu/~vmnih/data/ accessed on 1 September 2022). The data generated and analyzed during this study are available from the corresponding author by request.
